# Multiple giant coronary artery aneurysms combined with right coronary artery-pulmonary artery fistula: a case report

**DOI:** 10.1186/s12893-019-0547-z

**Published:** 2019-07-05

**Authors:** Jiayu Shen, Yang Zhou, Zhi Fang, Jia Hu

**Affiliations:** 1 Department of Cardiovascular Surgery, West China Hospital of Sichuan University, No.37 Guo Xue Alley, Chengdu, Sichuan 610041 People’s Republic of China; 2 Department of Cardiothoracic Surgery, People’s Hospital of Leshan, No.238 Bai Ta Street, Leshan, 614000 China

**Keywords:** Coronary artery aneurysm, Pulmonary artery fistula, Restrictive cardiac dysfunction

## Abstract

**Background:**

The combination of multiple giant coronary artery aneurysms (CAAs) and right coronary artery (RCA) to pulmonary artery (PA) fistula is extremely rare and the patients with CAAs may suffer from several fatal complications. We herein describe a 60-year-old female with hemodynamic instability who was diagnosed with multiple giant CAAs combined with RCA-PA fistula.

**Case presentation:**

The patient, a 60-year-old female, presented to the emergency room because of progressive exertional chest distress and fatigue. The transthoracic echocardiography (TTE), coronary computed tomography angiography (CTA) and invasive coronary angiography confirmed the existence of multiple giant CAAs and RCA-PA fistula. Laboratory examinations for systemic vasculitis and infectious diseases demonstrated no abnormalities and work-up for childhood and family history were negative. We have performed a successful surgical treatment for this patient. The patient’s restrictive cardiac dysfunction was improved after debriding the advanced thrombi in aneurysm sac and ligating the fistulous vessel between the native RCA and PA. The postoperative pathologic examination of the aneurysmal wall revealed loss of smooth muscle cells in the media with local mucoid degeneration, no chronic inflammation, sclerosis and IgG4 were observed.

**Conclusions:**

The treatment decision-making process should depend upon the patients’ specific situations. Our case suggests the surgical intervention should be accepted as the preferred treatment for giant CAAs with restrictive cardiac dysfunction.

**Electronic supplementary material:**

The online version of this article (10.1186/s12893-019-0547-z) contains supplementary material, which is available to authorized users.

## Background

Aneurysmal coronary artery disease is defined as a localized luminal dilation measuring at least 1.5 times the diameter of the largest adjacent reference segment. Even though the giant CAA generally refers to a dilatation>4 times the diameter of a reference vessel, the universally accepted definition of that term is still undetermined [1]. Even though the estimated incidence of CAAs is 0.3–5% in patients undergoing coronary artery angiography and is continuously increasing due to upgrade of imaging technology, giant CAA remains rare with an estimated incidence of 0.02% [2–4]. In adults, atherosclerosis is the most common cause for CAA, which accounts for 50% of all cases. Kawasaki disease, a generalized vasculitis of unknown etiology which occurs in children, also accounts for nearly 10% [5]. Patients with CAAs may suffer from fatal complications such as myocardial infraction and CAA rupture. The location and size of the CAA can also affect the patients’ clinical symptoms [6]. Despite the increasingly use of less invasive percutaneous techniques in selected patients nowadays, surgical correction is generally accepted as the preferred treatment for giant CAAs. We herein describe a successful treatment for a patient with severe heart failure who was diagnosed with multiple giant CAAs combined with RCA-to-PA fistula.

## Case presentation

**Fig. 1:**
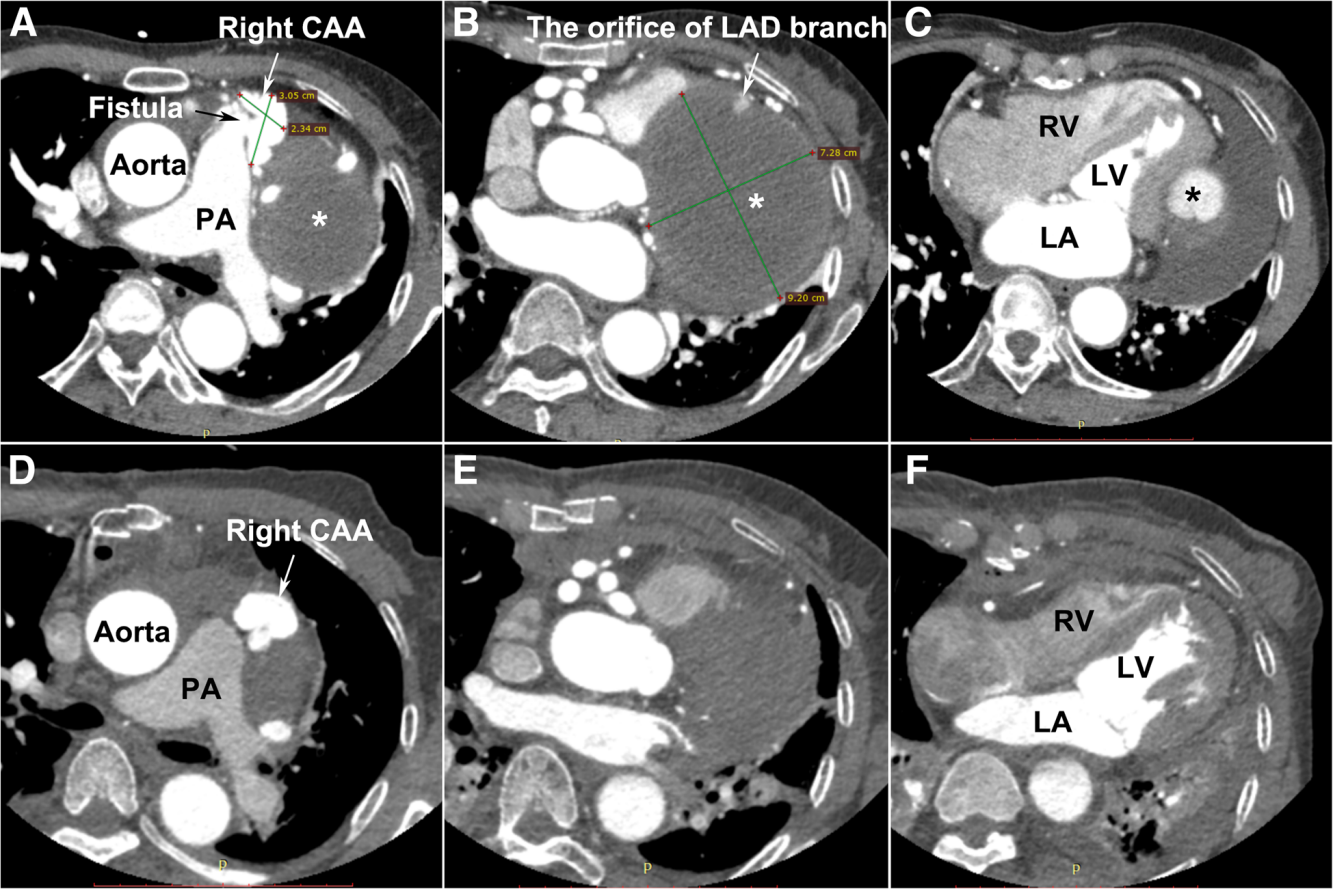
The transverse view of coronary CTA: **a** Giant right CAA with RCA-PA fistula; **b** and **c** left CAA, compressing the LV wall; **d**, **e** and **f** Postoperative CTA demonstrated no RCA-to-PA shunting and the oppression of the giant left CAA to left ventricle had been released. The diameter of left CAA decreased significantly. * CAA, coronary artery aneurysm; PA, pulmonary artery; LAD, left anterior descending coronary artery; LV, left ventricle; RV, right ventricle; CTA, computed tomography angiography

**Fig. 2:**
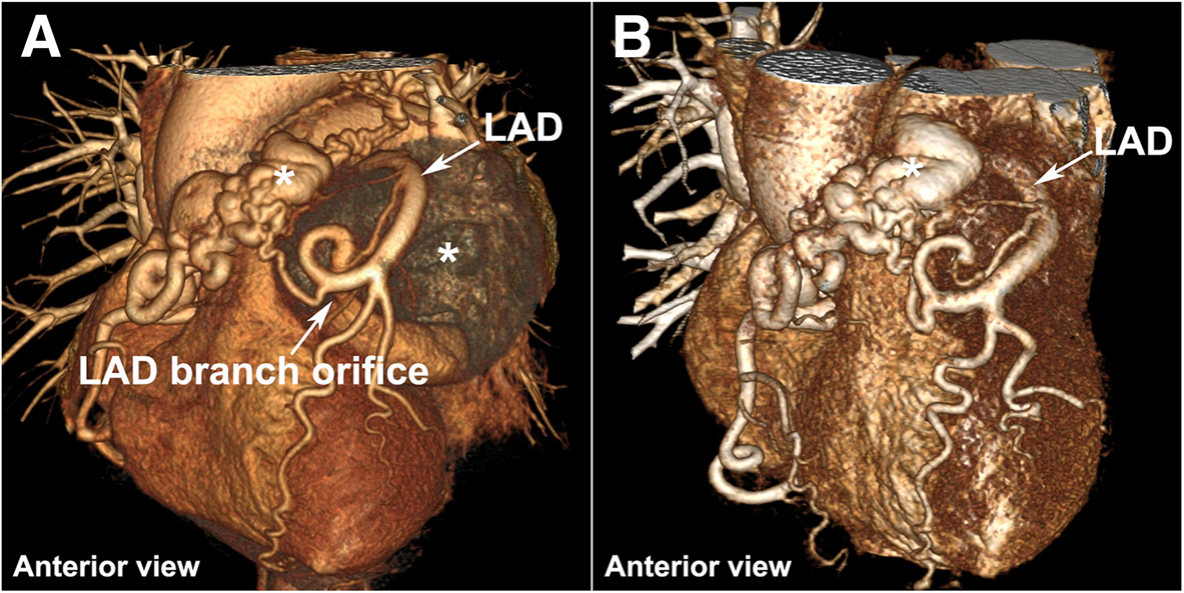
Anterior views of perioperative three-dimensional volume-rendered CTA. **a** Preoperative; **b** Postoperative. LAD, left anterior descending coronary artery; CTA, computed tomography angiography; *, coronary artery aneurysm


A 60-year-old female presented to the emergency room for progressive exertional chest distress and fatigue. The patient experienced paroxysmal nocturnal dyspnea and orthopnoea 2 days before admission. On admission, the physical examination revealed body temperature of 36.8 °C, heart rate 105 beats/min with sinus rhythm, respiratory rate 35 per minutes, blood pressure 85/64 mmHg and fine rales can be heard at bilateral lower lobes. Laboratory examinations for systemic vasculitis and infectious diseases demonstrated no abnormalities and work-up for childhood and family history were negative. TTE showed a giant circumscribed echolucent mass with suspicious intra-cavity thrombus compressing left ventricle, although the global ejection fraction (EF) was maintained at 54%. An abnormal communication between the RCA and PA trunk was also identified (see Additional file 1), and the Qp/Qs ratio was 2.5. A coronary CTA scan performed with a 16-detector row confirmed an extensive right CAA (3.05 × 2.34 cm in short-axis) with RCA-PA fistula (Fig. 1a) and a partially thrombosed giant aneurysm (9.20 × 7.28 cm in short-axis) arising from the branch of the left anterior descending (LAD) coronary artery, oppressing the left ventricle (Figs. 1b, c and 2a). The results of invasive coronary angiography correlated with the previous findings by CTA and TTE (see Additional file 2).

**Additional file 1:** Preoperative TTE revealed the compression of CAA to left ventricle and an abnormal communication between the RCA and PA trunk was also identified by transthoracic echocardiography. (MP4 9262 kb)



**Additional file 2:** The coronary angiography. (MP4 31754 kb)


**Fig. 3:**
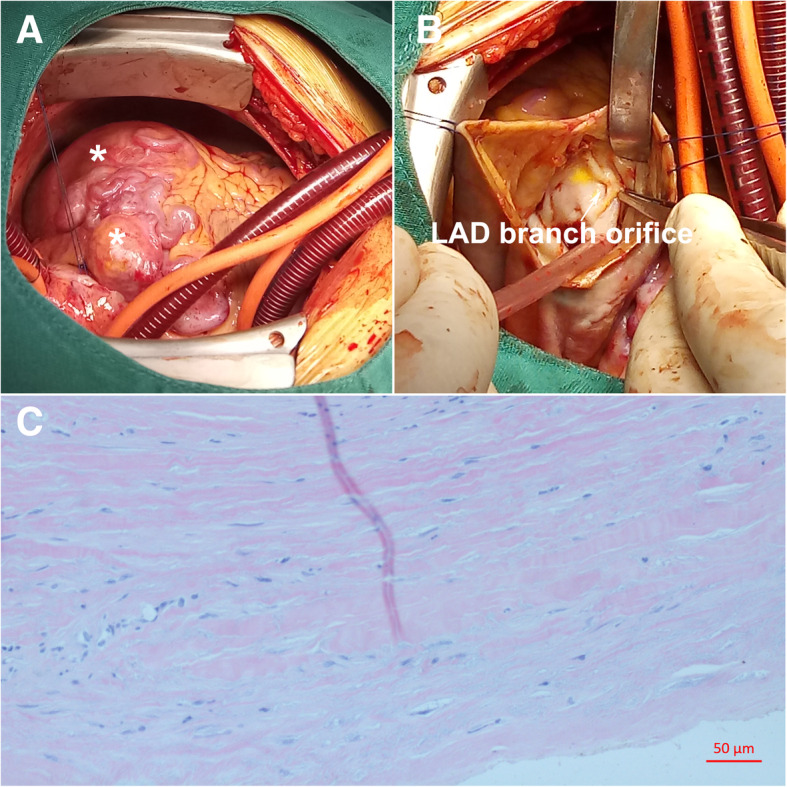
Intraoperative and pathological findings: **a** Multiple giant CAAs compress the left and right ventricular wall and extend toward the apex. **b** The orifice of one of the left CAA branches; **c** Pathologic examination of the aneurysmal wall revealed loss of smooth muscle cells in the media with local mucoid degeneration. * CAA, coronary artery aneurysm; LAD, left anterior descending coronary artery


Owing to the patient’s clinical status deteriorated continuously, the patient underwent surgical intervention under cardiopulmonary bypass to improve the hemodynamic instability due to restrictive cardiac dysfunction. Intra-operatively, a giant left CAA was found to be encasing the left and right ventricular wall and extending toward the apex (Fig. 3a). The fistulous vessel between the native RCA and PA was mobilized and ligated while we left the right CAA as it was before surgery. The left aneurysmal sac was opened and multiple hemorrhagic laminated thrombi were debrided completely. The orifice of the aneurysm connecting to one of the LAD branches was identified and closed with interrupted 5–0 prolene sutures (Fig. 3b). The patient weaned off bypass successfully and received lifelong antiplatelet therapy postoperatively. Postoperative TTE and CTA revealed no RCA-to-PA shunting or any flow in the residual aneurysmal sac and the compression of the giant left CAA imposed on the left ventricle had been obviated (Figs. 1d, e, f and 2b; Additional file 3). The patient experienced an uneventful postoperative course and discharged with improved heart function (EF: 68%, NYHA II). The pathologic examination of the aneurysmal wall revealed loss of smooth muscle cells in the media with local mucoid degeneration (Fig. 3c), no chronic inflammation, sclerosis and IgG4 were observed.

**Additional file 3:** Postoperative TTE revealed the decompression of ventricular wall and no residual RCA-to-PA shunting. (MP4 5651 kb)


## Discussion and conclusion

Multiple giant CAAs is extremely unusual in adults, extremely when it combined with RCA-to-PA fistula, which is a relative uncommon congenital heart malformation.

Although most CAAs are asymptomatic, some patients can present with angina pectoris, myocardial infarction, hemopericardium, tamponade, severe heart failure or sudden death [6]. Patients with giant CAAs can also present with superior vena cava syndrome or restrictive cardiac dysfunction, depending on the location and size of the CAA [7]. In this case, RCA-to-PA fistula not only resulted in the formation of the right CAA, but also contributed to the pulmonary hyperemia due to the left-to-right shunt. Meanwhile, the extremely huge left CAA restricted the diastole of the left ventricle and contributed to the hemodynamic abnormality due to restrictive cardiac dysfunction.

The exact pathogenesis of CAA remains to be fully understood. Several studies suggested that the essential component in the formation of CAA is an abnormal vessel media that may be secondary to an extension of the intimal arteriosclerosis process [4]. And several uremia-associated factors and disturbances of the calcium-phosphate metabolism may enhance the rate of atherosclerosis [8]. In the present case, however, the laboratory tests for hyperglycemia, hyperlipidemia and uremia-associated factors were negative. Moreover, our patient showed no vascular calcifications or coronary artery stenosis according to the invasive coronary angiography. There exists no evidence that the growth of our patient’s giant CAAs have been accelerated by the atherosclerosis. Recently, IgG4-related systemic disease was established as a novel clinicopathologic entity that can manifest in multiple organs, including the cardiovascular system [9]. Several previous reports described continuously expanded CAAs with remarkable thickness of the periarterial tissue which were eventually recognized as IgG4-related disease [10, 11]. In our patient, by using serum and histological examinations, no evidence of the IgG4-related periarteritis involving coronary arteries was detected. Furthermore, several studies have also reported that some hereditary factors may contribute to the formation of CAAs [12, 13]. However, according to the family history and physical examination, no potential genetic disorders was found in our patient’s family history and physical examination.

There is no consensus on the optimal management strategy for giant CAAs right now. Even though some studies recommend that the management of CAA varies according to the etiology, size and symptoms. Surgical correction, however, remains the mainstream treatment strategy for giant CAAs [14]. In the absence of surgical correction, asymptomatic CAAs can be managed with antiplatelet or antithrombotic treatment (or both), in order to reduce the risk of distal embolization. Despite the lack of substantial evidence-based results, anticoagulation and antiplatelet therapy do show positive effects in patients with Kawasaki disease [15, 16]. Less invasive techniques relying on using covered or uncovered stent also seem promising in curing CAAs in selected patients, while the long-term outcomes require further investigation [17]. Moreover, some studies argued that the percutaneous approaches to CAA exclusion seems reasonable for aneurysms greater than 5 mm but less than 10 mm [18]. Surgical interventions, such as aneurysm ligation with distal bypass grafting, isolate CABG, aneurysm plication and saphenous vein patch repair of the aneurysm, are generally accepted as the preferred treatment for giant CAA with any symptoms. A specific surgical approach must be selected in accordance with the size and anatomy of the aneurysm to perform safe and effective corrections, and the closure of the fistula is also mandatory if giant CAAs are combined with a fistula [19]. In this patient, the left giant CAA contributed to the restricted cardiac dysfunction and the left-to-right shunting of the RCA-to-PA fistula aggravated the pulmonary congestion. Owing to the left giant CAA originates from a branch of LAD and no signs of myocardial ischemia can be detected according to coronary angiography, we completely debrided thrombi in the left CAA sac and subsequently closed the proximal orifice, in order to relieve its oppression to left ventricle. As for the diffuse and extensive right CAA, surgical resection combined with CABG could be an effective and reasonable strategy. However, the poor preoperative condition of this patient prevented us from performing these time-consuming procedures. Moreover, the main trunk of the RCA was angiographically normal, and therefore we decided to merely ligate the RCA-PA fistula and leave the right CAA untreated. In order to prevent further thrombosis in delated coronary arteries, lifelong antiplatelet therapy is mandatory. The follow-up coronary CTA and TTE demonstrated no abnormalities.

In conclusion, we have reported a successful surgical treatment of extremely rare multiple giant CAAs combined with RCA-PA fistula in a patient with hemodynamic instability. Our practice indicates that the surgical intervention could be a referred choice in this circumstance.

## Data Availability

All data generated or analyzed during this study are included in this published article.

## Abbreviations

CAA: Coronary artery aneurysm
CABG: Coronary artery bypass grafting
CTA: Computed tomography angiographic
EF: Ejection fraction
LAD: Left anterior descending
NYHA: New York Heart Association
PA: Pulmonary artery
RCA: Right coronary artery
TTE: Transthoracic echocardiography
